# Auranofin and its analogs as prospective agents for the treatment of colorectal cancer

**DOI:** 10.20517/cdr.2021.71

**Published:** 2022-01-04

**Authors:** Lara Massai, Damiano Cirri, Tiziano Marzo, Luigi Messori

**Affiliations:** ^1^Department of Chemistry, University of Florence, Sesto Fiorentino 50019, Italy.; ^2^Department of Chemistry and Industrial Chemistry, University of Pisa, Pisa 56124, Italy.; ^3^Department of Pharmacy, University of Pisa, Pisa 56126, Italy.; ^4^CISUP - Centre for Instrumentation Sharing (Centro per l’Integrazione della Strumentazione Scientifica), University of Pisa, Pisa 56126, Italy.; ^5^University Consortium for Research in the Chemistry of Metal ions in Biological Systems (CIRCMSB), Bari 70126, Italy.

**Keywords:** Auranofin, CRC, colorectal cancer, metallodrugs, anticancer agents, gold, gold-based drugs

## Abstract

Today colorectal cancer (CRC) is one of the leading causes of cancer death worldwide. This disease is poorly chemo-sensitive toward the existing medical treatments so that new and more effective therapeutic agents are urgently needed and intensely sought. Platinum drugs, oxaliplatin in particular, were reported to produce some significant benefit in CRC treatment, triggering the general interest of medicinal chemists and oncologists for metal-based compounds as candidate anti-CRC drugs. Within this frame, gold compounds and, specifically, the established antiarthritic drug auranofin with its analogs, form a novel group of promising anticancer agents. Owing to its innovative mechanism of action and its favorable pharmacological profile, auranofin together with its derivatives are proposed here as novel experimental agents for CRC treatment, capable of overcoming resistance to platinum drugs. Some encouraging results in this direction have already been obtained. A few recent studies demonstrate that the action of auranofin may be further potentiated through the preparation of suitable pharmaceutical formulations capable of protecting the gold pharmacophore from unselective reactivity or through the design of highly synergic drug combinations. The perspectives of the research in this field are outlined.

## INTRODUCTION

Colorectal cancer (CRC) is one of the most common cancers in Western countries and is a major worldwide health problem being one of the primary causes of cancer death^[1]^. Advances in the diagnosis and treatment of CRC brought a major effect in the management of this disease. Notably, in the last decades, screening programs and early diagnosis, together with new therapeutic options, allowed reducing CRC mortality considerably^[2]^. Despite this progress and the remarkable increase of knowledge about CRC biology and treatment, metastatic cases are still associated with a poor prognosis. In fact, the percentage of survival drops from ~65% to ~10% in the presence of metastatic CRC disease^[3]^. At least half of patients with colorectal cancer develop metastases^[4]^, resulting in a poor outcome. The primary site for metastasis development is liver, representing the only site of metastasis in about one third of patients. Other common organs for distant metastases are lung, peritoneum, bone, brain, and spleen^[4,5]^. In CRC patients, the first therapeutic option is surgical resection, but the role of adjuvant chemotherapy (and chemoradiotherapy), in terms of disease-free survival and overall survival, is well recognized^[6,7]^.

CRC is reputed as poorly chemo-sensitive, and for more than 30 years fluorouracil has been the reference drug. Fluorouracil can be administered both through continuous infusion line and through bolus administration. Recent reports evidenced however no significant differences in terms of prolonged survival rate^[8,9]^. Many efforts have been made to improve these results. Biochemical modulation of fluorouracil is one of the most interesting approaches to increase the therapeutic index of this compound. In metastatic disease confined to the liver, locoregional therapy through implantable pumps may be taken into account as well^[10]^.

Until today, the standard therapy for CRC has relied on fluorouracil plus levamisole and/or calcium folinate (folinic acid)^[6]^. More recently, other treatment options have been introduced in the clinical practice. At present, there is increasing attention for the role of monoclonal antibodies (Mab) in CRC therapy. Among the various Mabs approved so far for CRC treatment, bevacizumab, panitumumab, and ramucirumab are the most commonly used^[11]^. Other clinical strategies rely on portal vein infusion of fluorouracil, alone or in combination with systemic therapy^[12]^. In rectal cancer, the best results are achieved by combining radiotherapy and chemotherapy. Recent reports support the use of induction or consolidation chemotherapy before surgery in locally advanced forms as well as total neoadjuvant treatment as the best therapeutic options^[13]^. In advanced colorectal cancer, a standard treatment has not been established yet, and different therapeutic options can be proposed according to the tumor staging and features. Typically, the first-line chemotherapy relies on the FOLFOX (leucovorin, 5-fluorouracil, and oxaliplatin) or CAPOX (capecitabine and oxaliplatin) protocol alone or in combination with Mab. At variance, the second-line approach also relies on compounds such as irinotecan and raltitrexed now entered in the clinical practice with encouraging results. For instance, FOLFIRI-based (leucovorin, 5-fluorouracil, and irinotecan) regimens are a common clinical practice, even in combination with antiangiogenic agents^[14]^.

## PLATINUM DRUGS IN CRC TREATMENT

Pt drugs play an important role in CRC treatment. Oxaliplatin is the only platinum anticancer drug in the clinical use for colorectal cancer, while cisplatin is not effective in the treatment of this tumor. The resistance to cisplatin in CRC is determined by several factors mostly related to the processing of cisplatin-induced DNA lesions by various biological actors. The tumor suppressor protein p53 is a key player in the cellular response to cisplatin. It is responsible for controlling the cascade of events leading to cell cycle arrest/repair or apoptosis through transcription and activation of numerous p53-dependent DNA damage response genes. Accordingly, deactivation of p53 in CRC is involved in cisplatin chemoresistance. In this view, the DNA mismatch repair (MMR) process has a relevant role as well. Indeed, cisplatin-induced lesions are recognized by MMR. Conversely, oxaliplatin-induced lesions to DNA are not recognized. As a consequence, oxaliplatin shows cytotoxic and anticancer effects that are basically independent of the MMR process^[15,16]^. However, beyond the formation of adducts with DNA, which are extensively recognized as a key step for the pharmacological activity of platinum-based anticancer drugs^[17,18]^, the different therapeutic indication between cisplatin and oxaliplatin was discussed by Bruno *et al*.^[19]^ in a seminal paper published in 2017. In this article, the authors reported on the mechanisms behind the anticancer effects of platinum-based drugs. Although it is not clear to what extent the effect of DNA-independent pathways contributes to the anticancer profile compared to the toxic effect of DNA adducts formation, the above authors pointed at the ribosome biogenesis stress induced by oxaliplatin as a likely mechanism also contributing to the overall pharmacological activity. This finding is relevant because might be involved in the distinct clinical implementation of oxaliplatin compared with cisplatin^[19]^.

As outlined above, Pt compounds, and oxaliplatin in particular, play a major role in the current medical treatment of CRC. Although being featured by a higher tolerability compared with cisplatin, oxaliplatin manifests a few relevant limitations such as relevant systemic toxicity and the frequent insurgence of resistance that may lead to eventual treatment failure. These limitations imply that the discovery of new drugs for colorectal cancer is absolutely mandatory and urgent. Here, we analyze specifically the chances of discovering new effective anticancer agents within the field of metal-based drugs.

## METAL BASED DRUGS AS A SOURCE OF NOVEL ANTICANCER AGENTS CAPABLE OF OVERCOMING DRUG RESISTANCE: THE CASE OF GOLD COMPOUNDS

Platinum-based compounds are very effective only against a relatively limited number of tumor types and manifest at the same time some severe side effects (e.g., gastrointestinal toxicity, nervous system toxicity, and bone marrow suppression) that heavily limit their use^[20-22]^. In addition, intrinsic and acquired drug resistance may greatly reduce the efficacy of platinum drugs with a consequent poor prognosis^[23]^. For this reason, intense efforts are warranted to explore and identify novel anti-tumor metallodrugs that may replace platinum compounds for specific therapeutic goals and for treatment improvement. Accordingly, many new metal compounds exploiting various transition metals have been prepared and evaluated, and some of them (e.g., gold, silver, copper, ruthenium, and other active metals) turned out to manifest very encouraging antitumor effects. In particular, coinage metals (especially Au and Ag) revealed a greater application potential as they are, on average, less toxic to humans than other transition metals. From a chemical point of view, gold compounds deserve particular attention owing to the unique position of gold in the periodic table, which ultimately leads to a larger electronegativity, a higher electron affinity, and a rich and peculiar redox profile. Several gold compounds, including both gold(III) and gold(I) compounds [i.e., gold(I) carbene, gold sodium thiomalate, gold thiolates, or gold compounds with bipyridyl-type ligands], were thus considered for cancer treatment, and many of them were found to possess remarkable antiproliferative properties *in vitro* against several human cancer cell lines^[24]^.

Several studies have pointed out that gold compounds cause their outstanding cytotoxic effects by taking advantage of multiple molecular and cellular mechanisms. The most credited mechanisms involve inhibition of thiol-containing enzymes, especially thioredoxin reductase (TrxR)^[25-27]^, direct mitochondrial damage^[28-31]^, or alteration of DNA functions^[32,33]^, all of which may contribute importantly to the observed anticancer actions. Although no non-platinum metal compound has been approved so far for clinical use, some gold drug candidates are being actively investigated. A few gold compounds have shown very promising results in preclinical research^[34]^, and two of them even reached clinical trials.

## AURANOFIN AND ITS ANALOGS AS ANTICANCER AGENTS: CHEMICO-BIOLOGICAL ASPECTS AND MECHANISTIC INFERENCES

**Figure 1:**
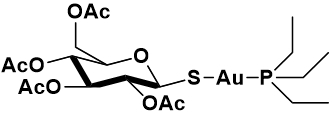
Chemical structure of auranofin.

In the field of gold-based drugs, auranofin (AF) undoubtedly occupies a pivotal position. From the chemical point of view auranofin, i.e., [2,3,4,6-tetra-o-acetyl-l-thio-β-d-glyco-pyrano-sato-S-(triethyl- phosphine)-gold(I)], is a mixed ligand gold(I) complex with a linear geometry having a triethylphosphine molecule and a thioglucose derivative as gold(I) ligands [Figure 1].

Recently, various research groups have found that auranofin^[35,36]^, beyond its known anti-inflammatory actions, also exhibits prominent anticancer, antibacterial, and antiparasitic properties^[37-43]^. Accordingly, during the past few years, auranofin has attracted a lot of attention in the medicinal chemistry scientific community as a prospective anticancer and anti-infective agent on the ground of drug repurposing strategies. As a matter of fact, AF has entered several different clinical trials as an anticancer, antiviral, or antiparasitic drug (ClinicalTrials.gov Identifier: NCT02089048, NCT01747798, NCT03456700, NCT01419691, NCT02063698, NCT02736968, NCT01737502, NCT02961829, NCT02770378, NCT03975790, and NCT01557348).

Auranofin was initially prepared in the late 1970s and found to manifest remarkable antiarthritic properties. Owing to its favorable pharmacological profile, auranofin was eventually approved for clinical use against rheumatoid arthritis in 1985, although in the absence of a precise understanding of its mode of action.

Moreover, it was found that auranofin was able to inhibit the growth of tumor cells *in vitro *and arrest the growth of an *in vivo* model tumor (leukemia P388) in mice. In fact, Mirabelli *et al*.^[44]^, starting from auranofin and changing systematically the phosphine ligand, the sulfur ligand, or both ligands, obtained 62 distinct Au(I) complexes. These novel complexes were tested both *in vitro*, against B16 melanoma and P388 leukemia, and *in vivo*, against mouse P388 intraperitoneal leukemia, with encouraging results. Mechanistically, it could be ascertained that auranofin behaves as a prodrug capable of releasing its two originals ligands; in any case, it is well documented that the thiosugar ligand is a better leaving group than the phosphine and is the first ligand to be released. The resulting empty coordination position on the gold(I) center becomes available for coordination to biomolecules. Typically, gold compounds with the general formula Et_3_PAuX manifest relevant and roughly similar anticancer profiles. This finding implies that the thiosugar ligand is not fundamental for the cytotoxic activity and that the [Et_3_PAu]^+^ moiety is likely to be the true pharmacophore. The role of the X ligand is probably related to the cellular uptake: a more lipophilic nature of the ligand might determine a more favorable pharmacokinetic and biodistribution profile.

**Figure 2:**
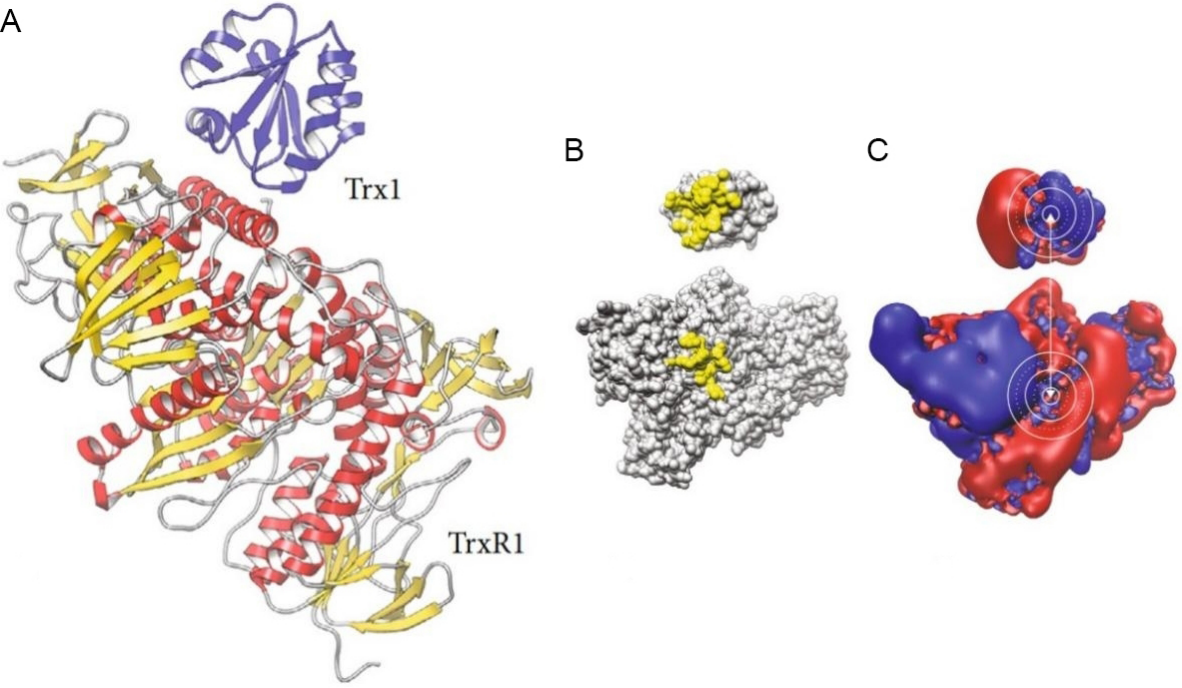
(A) Cartoon representation of the secondary structures of human thioredoxin-1-thioredoxin reductase 1 (Trx1-TrxR1) complex PDB: 3qfa. (B) The contact patches with direct molecular contacts are highlighted in yellow. (C) The isosurfaces of the electrostatic potential are depicted in blue (positive) and red (negative). The active site cysteinyl residues and interaction surfaces in the immediate contact area in both proteins are encircled in white lines. Reproduced and adapted from Hossain *et al*.^[47]^ under the Creative Commons Attribution License 4.0 International (CC BY 4.0).

The chemical behavior of auranofin and its reactions with biomolecules have been intensely investigated. The reaction of auranofin with biomolecules has now been studied in detail: it could be established that auranofin binds proteins tightly by forming strong coordinative bonds to free cysteine or selenocysteine residues^[45,46]^. This type of reactivity may well account for its molecular mechanisms; for instance, there is now a general consensus that the tight binding of auranofin to the free selenocysteine group in the active site of TrxR is primarily responsible for its relevant actions at the cellular level, eventually leading to severe intracellular redox dysregulation and associated apoptotic cancer cell death [Figure 2].

However, the interpretation of the mechanism of the anticancer actions of auranofin is still controversial and not conclusive. Although thioredoxin reductase inhibition is believed to be a central trait of its mode of action, other likely targets have been proposed and partially validated. As a matter of fact, some interesting proteomics data highlight, upon auranofin treatment, a few differentially expressed proteins belonging to different cellular processes, namely cell redox homeostasis, metabolism, and cell structure. Specifically, the main altered proteins were peroxiredoxins 1 and 6, linked to cell redox balance; triosephosphate isomerase 1, which plays a key role in the glycolysis and gluconeogenesis pathways; ezrin, essential for cell structure and cell migration; and the heterogeneous nuclear ribonucleoprotein H, whose increased cleavage leads to the caspase 3 activation triggering apoptosis^[24-48]^.

In addition, a few recent studies suggested that AF produces important immunomodulatory effects^[49]^. In particular, it was found that AF induces ICD (immunogenic cell death) in cancer cells as a consequence of endoplasmic reticulum stress and reactive oxygen species (ROS) production. Notably, Freire Boullosa *et al*.^[50]^ showed a significant increase in ICD-related damage-associated molecular patterns and maturation in dendritic cells following AF treatment in mutant p53 NSCLC *in vitro*. Apparently, these effects are mediated by the immunosuppressive TGF-β cytokine. TGF-β plays a major role in immunosuppression within the tumor microenvironment through the prevention of immune infiltration into tumor tissue and promotion of tumor cell proliferation. The cooperation between AF and anti-PD-L1 therapy in triple-negative breast cancer mouse model further supports the use of AF as an immunomodulating agent^[42]^. These findings are in accord with some early observations on the immunomodulatory properties of AF^[49]^.

Overall, these arguments demonstrate that auranofin, as already hypothesized for this compound as well as other gold(I) compounds, possesses a multi-target mode of action; indeed, auranofin interacts in cells and blood with several targets, mainly proteins with key functions, involving multiple cellular pathways and altering different biological networks.

## AURANOFIN AND ITS ANALOGS SHOW PROMISE FOR COLORECTAL CANCER TREATMENT: *IN VITRO *EVIDENCE

**Figure 3:**
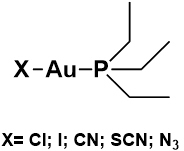
Chemical structures of auranofin analogs bearing different ligands in place of thiosugar moiety.

The favorable chemical and pharmacological profile of auranofin as a prospective anticancer agent prompted researchers to synthesize and characterize some auranofin derivatives and assess their action *in vitro* against a few CRC cell lines. In particular, in recent studies, five analogs of auranofin were prepared where the thiosugar ligand was substituted by different anionic ligands (these compounds are depicted in Figure 3)^[51,52]^. The synthesis of these analogs is rather straightforward and starts from the commercially available Et_3_PAuCl. More precisely, the iodide analog can be prepared through a simple chloride-displacing reaction carried out with an excess of potassium iodide^[51]^; the cyanide derivative can be synthesized reacting the two ionic species K[Au(CN)_2_] and [Au(PEt_3_)_2_]Cl in a biphasic reaction^[52]^; and the thiocyanate and azido derivatives can be prepared by reacting the Et_3_PAuCl species, respectively, with potassium thiocyanate and sodium azide after its activation with silver nitrate^[52]^.

Then, the antiproliferative properties of two of these analogs, i.e., Et_3_PAuCl and Et_3_PAuI, as well as auranofin itself were comparatively assayed *in vitro *against four representative colorectal cancer lines, i.e., HCT8, HCT116, HT29, and Caco2, and two healthy cell lines, i.e., HDF (human fibroblast, adult) and HEK293 (human embryonic kidney), by using the 3-(4,5-dimethylthiazol-2-yl)-2,5-diphenyltetrazolium bromide (MTT) test^[51]^. As displayed in Table 1, all tested compounds produced very notable cytotoxic effects on all the selected CRC cell lines with half-maximal inhibitory concentration (IC_50_) values always falling in the 100-700 nM range. Et_3_PAuI was slightly less cytotoxic than the other two gold complexes. In line with expectations, the presence of the thiosugar ligand turned out not to be an essential feature for the cytotoxic action. Moreover, by considering the close similarity in the measured IC_50_ values, it can be inferred that the cellular uptake of the three compounds should not be very different. Remarkably, when measuring the cytotoxic effects on two healthy cell lines, HDF human fibroblast cells (adult) and HEK293 human embryonic kidney, no appreciable cytotoxic effects were detected for the three study complexes in the concentration range 0-5000 nM. The latter finding is a good index of selectivity for cancer cells.

**Table 1:** IC_50_ values (nM) determined for Et_3_PAuI, AF, and Et_3_AuP-Cl (24 h incubation)

**Complex**	**HCT8**	**HCT116**	**HT29**	**Caco2**	**HDF**	**HEK293**
**AF**	132 ± 16	180 ± 17	359 ± 35	465 ± 53	> 5000	> 5000
**Et_3_PAuCl**	105 ± 11	154 ± 22	122 ± 15	560 ± 93	> 5000	> 5000
**Et_3_PAuI**	260 ± 28	290 ± 36	318 ± 90	706 ± 232	> 5000	> 5000

The results are reported as the average value for three independent experiments ± standard deviation. Reproduced and adapted with permission from Marzo *et al*.^[51]^. IC_50_: Half-maximal inhibitory concentration; AF: auranofin.

Afterwards, since TrxR is reputed to be a primary target for auranofin, the inhibitory potencies of the three gold compounds against this enzyme were analyzed comparatively. The results are summarized in Table 2. Interestingly, the obtained IC_50_ values for TrxR inhibition are roughly consistent with those determined for the cytotoxic effects in CRC cell lines [Table 1]. This probably implies that the observed cytotoxic effects are somehow linked to the ability of these gold complexes to inhibit TrxR. Moreover, the present results confirm that Et_3_PAuCl is not only the most potent cytotoxic agent but also the most potent TrxR inhibitor of the series; although the IC_50_ values measured for auranofin and Et_3_PAuI are only slightly higher, they still fall in the nanomolar range.

**Table 2:** Thioredoxin reductase activity assay. IC_50_ values (nM) were determined treating 2 U/L of TrxR with aliquots of AF, Et_3_AuPCl, and Et_3_PAuI (from 1 µM to 1 nM)

**Complex**	**IC_50_ (nM)**
AF	105 ± 17.3
Et_3_AuPCl	51.3 ± 8.5
Et_3_PAuI	193 ± 22.2

The results are reported as the average value for three independent experiments ± standard deviation. IC_50_ refers to 50% enzyme inhibition. Reproduced and adapted with permission from Marzo *et al*.^[51]^. IC_50_: Half-maximal inhibitory concentration; AF: auranofin.

Conversely, the three auranofin analogs in which the thiosugar was replaced with stronger ligands (i.e., Et_3_PAuCN, Et_3_PAuSCN, and Et_3_PAuN_3_) turned out to be completely inactive against HCT116 cell line for concentrations ranging up to 1000 nM. This observation strongly supports the occurrence of a reaction mechanism in which the anionic groups have to be displaced from the nucleophilic active site of TrxR (the SEC-CYS motif) to observe the pharmacological activity of the [Au(PEt_3_)]^+^ moiety^[52]^. In conclusion, the results of this study demonstrated that auranofin and its chloride, and the iodide analogs manifest potent cytotoxic effects *in vitro *against four selected CRC lines with the measured IC_50_ values always falling in the nanomolar range and no apparent cytotoxic effect on human fibroblast cell line and human embryonic kidney cells up to a 5 µM concentration. The TrxR activity assay revealed that both Et_3_PAuCl and Et_3_PAuI retain the potent inhibitory action of auranofin (nanomolar range), being consistent with their observed cytotoxic effects. Overall, these findings are consistent with the concept that TrxR remains the most probable and most relevant biomolecular target for these gold compounds. Moreover, these results, although obtained *in vitro* on cell cultures, support the idea that auranofin and its analogs are optimal drug candidates for further testing against more advanced and sophisticated CRC models. It should also be noted that the DNA-independent mode of action of AF and some of its analogs is a key aspect determining the high cytotoxicity toward *in vitro* CRC models. As an example, AF, Et_3_PAuCl, and Et_3_PAuI [Table 1] have been reported to exert anticancer effects significantly greater than cisplatin and even oxaliplatin on representative colorectal cancer cell lines [Table 3]^[51,52]^.

**Table 3:** The IC_50_ values (µM) determined for cisplatin and oxaliplatin (24 h incubation) against HT29 and HCT116 lines are also included as reference

**Complex**	**HCT116**	**HT29**
**Cisplatin**	21.96 ± 1.11	16.39 ± 1.10
**Oxaliplatin**	49.2 ± 0.9	19.7 ± 1.2

Reproduced and adapted with permission from Marzo *et al*.^[53]^ and Cirri *et al*.^[54]^. IC_50_: Half-maximal inhibitory concentration.

Based on the above considerations, it could be inferred that the ability of auranofin and some of its analogs to exert a greater anticancer activity in CRC lines than cisplatin and oxaliplatin might depend on the different mechanisms underlying the pharmacological effects. In fact, in contrast to oxaliplatin^[55]^, auranofin does not efficiently bind DNA, thus being its activity substantially unaffected by MMR, p53, and nucleotide excision repair functions. In fact, gold-based drugs produce the desired anticancer effects mainly through a DNA-independent mode, i.e., targeting specific enzymes such as the Trx system^[51,52]^.

## NEW PERSPECTIVES IN THE USE OF AURANOFIN AND ITS ANALOGS: ENCAPSULATION OF GOLD COMPOUNDS IN BIOCOMPATIBLE NANOPARTICLES

**Figure 4:**
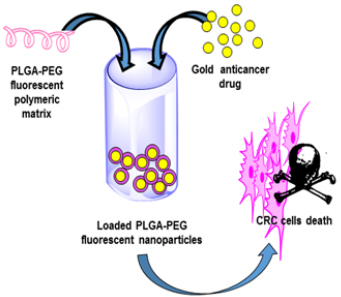
Schematic representation of the Et_3_PAuCl-loaded NPs tested against colorectal cancer models. Reproduced and adapted from Menconi *et al*.^[43]^ under the Creative Commons Attribution License 4.0 International (CC BY 4.0).

As stated above, Et_3_PAuCl is an auranofin derivative exhibiting very attractive biological and pharmacological properties. Similar to auranofin, Et_3_PAuCl possesses potent cytotoxic properties *in vitro* toward numerous cancer cell lines, thus being a promising anticancer drug candidate. In this frame, some investigators wondered whether Et_3_PAuCl encapsulation might lead to a better pharmacological profile, considering the expected reduction of unwanted side-reactions that are mainly responsible for the adverse effects and for drug inactivation. A reasonable option to achieve this goal consists in using biocompatible nanoparticles as nanocarriers to protect the gold complex from the biological environment. To achieve this goal, Menconi *et al*.^[43]^ exploited organic polyethylene glycol-poly lactic acid-co-glycolic acid (PLGA-PEG)-based nanoparticles of intermediate size, which could host a certain number of metallodrug’s copies into their hydrophobic core [Figure 4]. Et_3_PAuCl was encapsulated in these biocompatible PLGA-PEG nanoparticles, and the new formulation was evaluated in colorectal HCT116 cancer cells in comparison to free Et_3_PAuCl.

Notably, the encapsulated Et_3_PAuCl mostly retains the cellular actions of the free complex and causes even larger cytotoxic effects in CRC cells, through apoptosis and autophagy. Moreover, a large inhibition of two crucial signaling pathways, namely extracellular signal-regulated kinase (ERK) and protein kinase B (AKT), by the encapsulated form of Et_3_PAuCl, was clearly evidenced by the fact that this inhibition was not found in cells treated with the free drug. Overall, these results point out that encapsulation of Et_3_PAuCl in PLGA-PEG nanoparticles does not significantly affect the antiproliferative properties of this gold complex. However, some changes in the biological effects of the studied gold complex could be detected, which were specifically evidenced by the differential effects produced on the ERK and AKT signaling pathways. It would be of interest to extend such experimentation to appropriate *in vivo* models of CRC.

## NEW PERSPECTIVES IN THE USE OF AURANOFIN AND ITS ANALOGS: THE ROLE OF COMBINATION THERAPIES

Another valuable strategy in the use of gold compounds as anti-colorectal cancer agents is offered by the exploitation of so-called combination therapies. It is a common practice in cancer pharmacology to use anticancer drugs in association. Cocktails of drugs instead of single drugs are indeed very popular in current anticancer medical treatments for various reasons: (1) the application of lower concentrations of intrinsically toxic drugs with a narrow therapeutic index; (2) the opportunity to achieve a considerable synergism; and (3) the effective chance of reducing resistance insurgency. Combination therapy may also consist of the combination of an anticancer drug with a common non-cytotoxic drug that has nonetheless the important potential to modulate/enhance the cytotoxic effect of the first drug. This type of strategy was applied very recently by Han *et al*.^[56]^ to auranofin for the treatment of CRC. In detail, these authors performed a high-throughput screening of a library of 1280 FDA-approved clinical drugs in the search for compounds that might enhance the anticancer activity of auranofin *in vitro*. Surprisingly, they found that the anti-inflammatory drug celecoxib (CE), a cyclooxygenase 2 inhibitor, strongly potentiated the anticancer activity of AF^[56]^.

**Figure 5:**
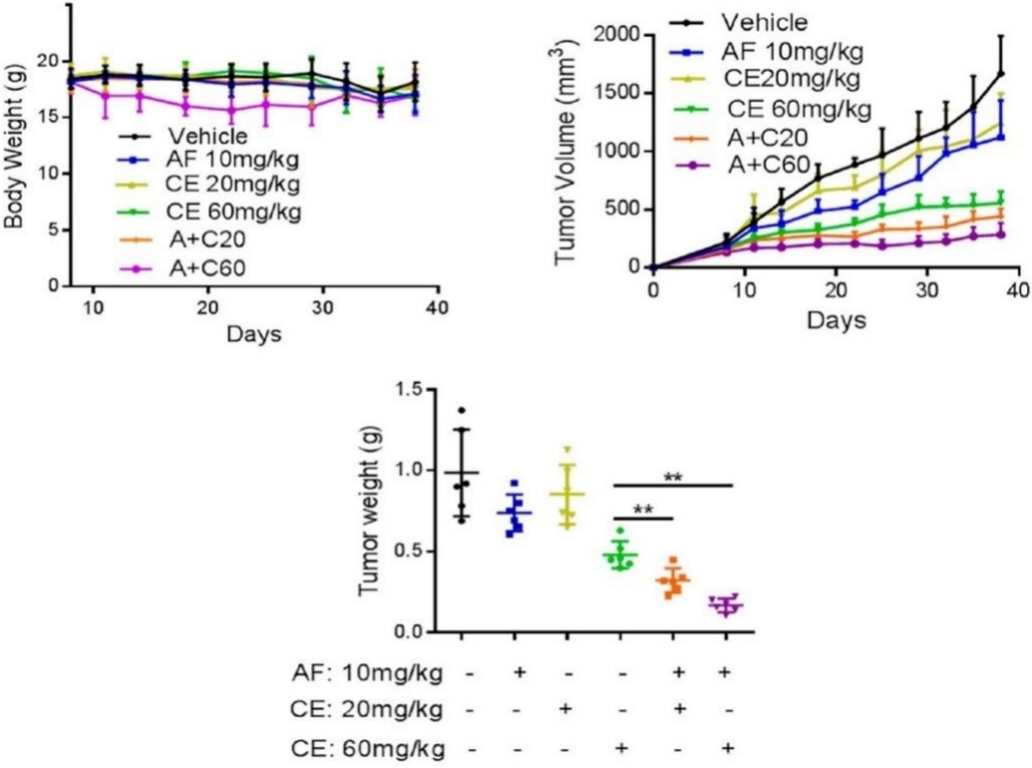
The effect of the combination of auranofin with celecoxib in mice. Athymic nude mice bearing DLD-1 xenografts were treated with the following drugs via oral injection (P.O.): olive oil (vehicle), AF 10 mg/kg, CE 20 mg/kg, CE 60 mg/kg, AF 10 mg/kg + CE 20 mg/kg (A + C20), and AF 10 mg/kg + CE 60 mg/kg (A + C60). Eight days after inoculation, the tumor size and body weight of mice from each group (six mice per group) were measured two times per week. The three panels show the body weight, tumor volume, and tumor weight during the treatment. Reproduced and adapted from Han *et al*.^[56]^ under the Creative Commons Attribution License 4.0 International (CC BY 4.0). AF: Auranofin; CE: celecoxib.

Notably, the promising *in vitro* results obtained for the AF + CE association were later supported by very encouraging *in vivo* results, as displayed in Figure 5. Since AF and CE are FDA-approved drugs that are used in the clinic, it is quite straightforward to translate the results of this study into an immediate clinical cancer treatment.

**Figure 6:**
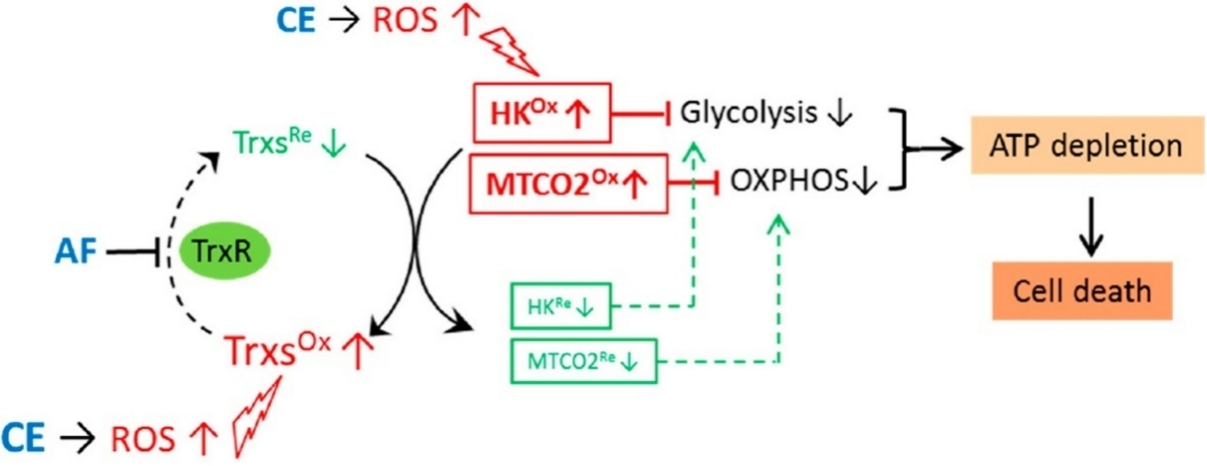
Synergy between auranofin and celecoxib against colon cancer *in vitro* and *in vivo* through a novel redox-mediated mechanism. CE induces ROS increase, which in turn causes oxidation of proteins (Trxs, HK, and MTCO2). AF inhibits TrxR, and thus keeps Trxs in oxidized form, which cannot reduce/repair the oxidized proteins (HK and MTCO2), leading to inhibition of both glycolysis and mitorespiration, ATP depletion, and cell death. Reproduced and adapted from Han *et al*.^[56]^ under the Creative Commons Attribution License 4.0 International (CC BY 4.0). CE: Celecoxib; ROS: reactive oxygen species; HK: hexokinase.

Mechanistically, the AF/CE combination induced severe oxidative stress, resulting in ROS-mediated hexokinase inhibition and disruption of mitochondrial redox homeostasis. Overall, these effects eventually caused a significant decrease of ATP generation. The CE-induced ROS increase together with AF-mediated inhibition of thioredoxin reductase determined a large shift of Trx2 to its oxidized form, producing a degradation of MT-CO2 (mitochondrially-encoded cytochrome C oxidase II) and a dysfunction of the electron transport chain (see Figure 6).

## CONCLUSIONS

CRC is the second most deadly cancer worldwide. Medical treatments for CRC are still largely insufficient and rarely curative; it follows that the inoperable metastatic disease results in most cases in patient’s death^[57]^. Metal-based drugs may play a significant and growing role in the therapeutics of CRC; oxaliplatin is already employed in the treatment of this disease, but its use is often limited by the insurgence of platinum resistance. Gold compounds are promising experimental anticancer drugs and might offer a valuable alternative to platinum drugs by overcoming resistance to Pt drugs^[58]^. Among the existing medicinal gold compounds, auranofin and its analogs - given the ease of their repurposing - may be the most appropriate and obvious drug candidates for CRC^[59]^. Auranofin has indeed manifested relevant anticancer actions and already entered clinical trials for other types of cancer, in particular ovarian cancer and various hematological malignancies. Surprisingly, auranofin has been scarcely tested so far for CRC treatment. However, just a few years ago, it was demonstrated that AF is very effective *in vitro* against four representative CRC lines while being far less toxic for healthy cells, thus showing some degree of selectivity. Moreover, a few studies in the recent literature suggest new valuable strategies to improve the pharmacological profiles of AF and its analogs. We refer specifically to a couple of studies that delineate feasible strategies for therapeutic intervention^[43,56]^. In a first study, encapsulation of Et_3_PAuCl in PLGA nanoparticles proved to bring about some favorable pharmacological effects such as retention of the cytotoxic activity, attenuation of the general reactivity of the gold center, and expected reduction of the drug’s adverse effects. Alternatively, a second study showed that the anticancer action of AF in CRC may be greatly potentiated through appropriate drug combinations. In fact, a systematic screening procedure applied to a large library of 1280 FDA-approved drugs revealed a strong synergism between auranofin and the anti-inflammatory drug celecoxib. This synergism was well documented both *in vitro* and *in vivo*. In view of these initial yet very encouraging results, we propose that the testing of auranofin and its analogs toward suitable CRC models is further expanded and encouraged taking advantage of new pharmaceutical formulations and appropriate drug combinations. Additionally, extensive *in vivo* testing of these gold compounds against suitable animal models of CRC would be highly desirable at this stage to reinforce and validate the concepts presented here.

## DECLARATIONS

### Copyright

© The Author(s) 2022.
